# High Coverage and Utilization of Fortified Take-Home Rations among Children 6–35 Months of Age Provided through the Integrated Child Development Services Program: Findings from a Cross-Sectional Survey in Telangana, India

**DOI:** 10.1371/journal.pone.0160814

**Published:** 2016-10-03

**Authors:** Magali Leyvraz, James P. Wirth, Bradley A. Woodruff, Rajan Sankar, Prahlad R. Sodani, Narottam D. Sharma, Grant J. Aaron

**Affiliations:** 1 Global Alliance for Improved Nutrition, Geneva, Switzerland; 2 GroundWork, Fläsch, Switzerland; 3 TATA Trusts, New Delhi, India; 4 Indian Institute of Health Management Research, Jaipur, India; Universidade de Sao Paulo, BRAZIL

## Abstract

The Integrated Child Development Services (ICDS) in the State of Telangana, India, freely provides a fortified complementary food product, Bal Amrutham, as a take-home ration to children 6–35 months of age. In order to understand the potential for impact of any intervention, it is essential to assess coverage and utilization of the program and to address the barriers to its coverage and utilization. A two-stage, stratified cross-sectional cluster survey was conducted to estimate the coverage and utilization of Bal Amrutham and to identify their barriers and drivers. In randomly selected catchment areas of ICDS centers, children under 36 months of age were randomly selected. A questionnaire, constructed from different validated and standard modules and designed to collect coverage data on nutrition programs, was administered to caregivers. A total of 1,077 children were enrolled in the survey. The coverage of the fortified take-home ration was found to be high among the target population. Nearly all caregivers (93.7%) had heard of Bal Amrutham and 86.8% had already received the product for the target child. Among the children surveyed, 57.2% consumed the product regularly. The ICDS program’s services were not found to be a barrier to product coverage. In fact, the ICDS program was found to be widely available, accessible, accepted, and utilized by the population in both urban and rural catchment areas, as well as among poor and non-poor households. However, two barriers to optimal coverage were found: the irregular supply of the product to the beneficiaries and the intra-household sharing of the product. Although sharing was common, the product was estimated to provide the target children with significant proportions of the daily requirements of macro- and micronutrients. Bal Amrutham is widely available, accepted, and consumed among the target population in the catchment areas of ICDS centers. The coverage of the product could be further increased by improving the supply chain.

## Introduction

In the 1970s, the Government of India adopted a series of policies aimed at improving the health of the country’s population and reducing the prevalence of malnutrition. Among these policies was the National Policy for Children, which was adopted in 1974 to improve the development and health of children, as well as to increase access to food for children [1]. In 1975, in pursuance of this policy, a program called the Integrated Child Development Services (ICDS) was launched [2]. The purpose of ICDS is to provide a number of basic nutrition, health, and early child development services to children from birth to 6 years of age and to pregnant and lactating mothers [3, 4]. The services are provided through local centers, called *anganwadi* centers (AWCs), and include immunization, health care, preschool education, maternal education, family planning, referral services, vitamin A supplementation, iron-folic acid supplementation, provision of iodized salt, basic nutrition and child feeding education, and provision of supplementary food [5, 6].

According to India's Ministry of Women and Child Development [7], it is estimated that more than 1.3 million AWCs are operating in India, making ICDS one of the largest delivery platforms in the country. The ICDS program has been successful in many ways, but still faces a number of implementation and operational challenges [8]. Effective delivery of the services remains limited, especially in relation to promotion of appropriate complementary feeding. For example, results from a recent national survey indicated that about three-quarters of the children under 3 years of age living in an area covered by an AWC do not receive any supplementary food, despite this being part of the core package of services [3].

In response to this need, the Global Alliance for Improved Nutrition (GAIN) began supporting Andhra Pradesh Foods (AP Foods), a state government-owned food manufacturing company that was producing take-home rations for ICDS in 2010. GAIN supported AP Foods to produce Bal Amrutham, a porridge fortified with multiple micronutrients to be distributed as a take-home ration to children participating in the ICDS program [9]. The goals of the project were to increase the production capacity of AP Foods, upgrade the quality assurance and control systems, and improve the nutritional content and packaging of Bal Amrutham. Some of the accomplishments of the project include construction of a new manufacturing plant, increased production capacity, upgrade of the quality assurance and control systems, improvement of the packaging and product shelf life, removal of all trans fats, and addition of milk powder.

As GAIN’s support for the project ended in 2014, a state-wide survey was conducted to assess the project’s coverage and utilization. At about the same time, in June 2014, Andhra Pradesh was divided into two independent states, Telangana and Seemandhra, by the Andhra Pradesh Reorganization Act. The continued distribution of Bal Amrutham was ensured only in Telangana, where AP Foods is based, and therefore the survey was conducted in Telangana only.

The main objectives of the survey were to determine the coverage and utilization of the Bal Amrutham in areas of Telangana where the ICDS program was implemented, to assess the accessibility and utilization of the ICDS program, and to determine the associations between product coverage and other health and nutrition indicators. A secondary objective of the survey was to assess household coverage of iodized salt and the potential for rice fortification, the results of which are presented in another paper [10].

## Methods

### Product description

Bal Amrutham is distributed by the AWCs as 2 kilogram take-home ration bags once per month to children 6–35 months. The macro- and micronutrient contents of Bal Amrutham are shown in Table 1.

**Table 1:** Macro- and Micronutrient Composition of Bal Amrutham.

Component	Amount per 100 grams
Energy (kcal)	414
Protein (g)	11
Fat (g)	11
Iron (mg)	9.1
Vitamin A (μg)	202.5
Calcium (mg)	367
Vitamin B1 (mg)	0.6
Vitamin B2 (mg)	0.55
Vitamin C (mg)	15.3
Folic acid (μg)	22.1
Niacin (mg)	6.3

### Survey design, location, and population

A two-stage, stratified cross-sectional cluster survey was conducted from November to December 2014. The survey was designed to be representative of children between 0 and 35 months of age in the catchment areas of AWCs across the State of Telangana. Every AWC is intended to cover approximately 200 households, which corresponds to 1,000 total population and about 55 children between 6 and 35 months [8]. Each AWC was assigned to one of two strata, urban or rural (the rural stratum included AWCs classified as “tribal”), and 45 AWCs were randomly selected in each stratum. Each selected AWC provided a list of all the children between 0 and 35 months in its catchment area. To ensure that the list was accurate, the *anganwadi* workers (AWWs) also provided information on the catchment area of the AWC, and the data collection teams conducted a census of all children 0–35 months in the defined catchment area. For the second stage of sampling, 13 children aged 0–35 months were selected via simple random sampling. The primary caregiver (i.e., the person, female or male, who fed the child on most days) of the selected child was interviewed. If the child or his/her primary caregiver was absent at the time of the visit, a maximum of two repeat visits were made.

Although the target population of Bal Amrutham was children 6–35 months, all children 0–35 months of age were surveyed to permit the assessment of exclusive breastfeeding and the incorrect use of complementary foods among children under 6 months of age.

### Ethical clearance

Ethical clearance for the survey was obtained from the Institutional Review Board at the Institute of Health Management Research Ethics Committee (#IORG0007355). After being informed about the study, oral informed consent was sought from the primary caregiver for herself/himself and for the selected child and recorded by the interviewer on the consent form. Any caregiver or child diagnosed with severe acute malnutrition (based on mid-upper arm circumference [MUAC]) was referred to the nearest health facility.

### Survey instrument and indicator definitions

The questionnaire utilized GAIN’s Fortification Assessment Coverage Tool (FACT). The FACT contained various standardized modules and was used to collect information on the respondent’s socio-demographic status; water, sanitation, and hygiene practices; infant and young child feeding (IYCF) practices; women and child dietary diversity; and coverage of the ICDS program and the fortified take-home ration. Moreover, the MUAC of the child and caregiver were measured using a MUAC measuring tape.

Acute malnutrition was defined in children under 6 months of age as a MUAC of less than 115 mm, in children of 6–36 months of age as a MUAC of less than 125 mm, and in adult women as a MUAC of less than 230 mm. The household dependency ratio was calculated by dividing the number of household members under 15 years old and above 64 years old by the number of household members between 15 and 64 years old.

The multidimensional poverty index (MPI) is a composite indicator that is calculated from indicators on living standards, education, health, and nutrition, whereby 0 indicates no poverty and 1 indicates maximum poverty [11]. A household with an MPI score ≥ 0.33 was considered to be at risk for poverty. The Infant and Child Feeding Index (ICFI) was calculated according to Guevarra et al. [12] to assess the quality of IYCF practices in all children 0–35 months of age. Household food insecurity was determined by asking about the availability of two full meals per day for all household members during the full year [13]: If not all household members received two full meals per day during the whole year, then the household was categorized as at risk of food insecurity.

Using the approach from Tanahashi[14], four coverage measures were defined to characterize the different coverage and utilization levels of Bal Amrutham: “message coverage” (i.e., the caregiver has ever heard of Bal Amrutham), “contact coverage” (i.e., the caregiver has ever received Bal Amrutham for the index child), “any coverage” (i.e., the target child consumes Bal Amrutham sometimes or always), and “effective coverage” (i.e., the target child always consumes Bal Amrutham). In addition, the availability, accessibility, acceptability, and utilization of the ICDS program’s services were studied to assess whether the ICDS program in itself was related to the coverage of the product.

Two summary statistics were calculated for each coverage measure: “met need” (the proportion of children considered as at risk of poverty who are covered) and “coverage ratio” (CR) (the ratio of the coverage in children considered at risk of poverty to the coverage in children considered *not* at risk of poverty). CR values below 1 indicate poor targeting (i.e., coverage is lower in children at risk of poverty), and CR values above 1 indicate efficient targeting (i.e., coverage is higher in children at risk of poverty). A CR value of 1 indicates lack of targeting (i.e., coverage is equivalent in children both at-risk and non-at-risk of poverty). The contribution of Bal Amrutham to daily recommended intakes was estimated based on the recommended dietary allowances for Indian children [15].

### Statistical analysis

Before data were entered, each questionnaire was checked independently by three different supervisors to ensure completeness and correctness. Double data entry was conducted using CSPro (Version 5). Overall estimates were weighted to account for unequal probability of selection due to stratified sampling and the selection of primary sampling units with equal probability.

For categorical variables, the statistical significance of differences between subgroups was assessed using adjusted chi-square p-values. For continuous variables, statistical significance was assessed using adjusted student’s t-test and one-way analysis of variance (ANOVA). Additionally, multivariate regression analysis was conducted to assess independent associations between coverage and factors associated with coverage. Percent met need and CRs were calculated using a blocked weighted bootstrap estimation technique [16] with a total of 400 bootstrap replicates. P-values of less than 0.05 and CRs where the confidence interval did not include 1 were considered statistically significant. Data analyses were conducted using R (Version 3.1.0) and SPSS (version 21, IBM Corporation, Armonk, NY, USA).

## Results

### Characteristics of the survey sample

A total of 1,157 children were randomly selected, and 1,077 completed the survey (93.1% response rate). Among all the caregivers interviewed, nine were males and the rest were females. The characteristics of the population surveyed are shown in Table 2. The household dependency ratio indicates that there was close to one person in the working age range for each dependent person (defined as a person not in the working age range) in the household. Among the households surveyed, 23.2% were classified as at risk for poverty by the MPI.

**Table 2:** Survey Population Demographics and Household Characteristics.

Variable	N	Values^a^
**Household level**		
Household size (mean)	1,077	5.0 (4.9, 5.1)
Household dependency ratio (mean)	1,077	0.87 (0.80, 0.97)
Poor households (%)	1,039	23.2 (17.6, 29.9)
Electricity (%)	1,039	97.6 (95.0, 98.9)
Clean cooking fuel (%)	1,039	47.5 (41.1, 54.1)
Improved flooring (%)	1,039	82.0 (76.8, 86.2)
Safe drinking water source (%)	1,039	95.8 (91.6, 98.0)
Adequate toilet sanitation (%)	1,039	34.6 (28.6, 41.1)
One or more household members 5–14 years not currently attending school (%)	1,039	6.0 (3.7, 9.3)
Food insecure (%)	1,039	2.8 (1.7, 4.6)
**Caregiver**		
Age (mean years)	1,077	25.3 (24.5, 26.1)
≥ 5 years of schooling (%)	1,039	60.7 (52.6, 68.2)
Women with low MUAC^b^ (%)	1,039	34.4 (29.5, 39.7)
**Child**		
Age (mean months)	1,077	16.9 (16.1, 17.9)
Sex, female (%)	1,077	50.4 (45.3, 55.4)
Children with low MUAC^c^ (%)	1,039	8.3 (6.4, 10.8)

^a^ All values are mean (95% CI) or percentage (95% CI), as indicated.

^b^ Low MUAC is defined as MUAC below 230 mm

^c^ Low MUAC is defined as below 115 mm for children <6 months and below 125 mm for children 6–36 months. Missing values were imputed with age-group median MUAC

The IYCF practices of the surveyed population are presented in Table 3. Exclusive breastfeeding among children under 6 months of age and continued breastfeeding among children 6–23 months of age was high (89.1% and 82.7%, respectively). However, complementary feeding practices were found to be poor, especially dietary diversity, which was only about 47% of the children 6–35 months of age. IYCF practices were not significantly different between poor and non-poor households.

**Table 3:** Infant and Child Feeding Index Indicators^a^.

Variable	N	All	Poor^b^	Non-poor	P-value
ICFI score of children 6–35 months (mean)	903	4.82 (4.68, 4.95)	4.91 (4.71, 5.10)	4.78 (4.62, 4.95)	0.287
Children 6–35 months with optimal IYCF practices (%)^c^	869	29.2 (24.4, 34.5)	29.5 (20.9, 39.8)	29.1 (24.0, 34.8)	0.942
Children 0–35 months with optimal IYCF practices (%)^c^	1,039	38.7 (33.5, 44.1)	38.2 (28.9, 48.4)	38.8 (33.7, 44.3)	0.896
Children 6–23 months with continued breastfeeding (%)	571	82.7 (76.7, 87.4)	82.3 (65.8, 91.9)	82.8 (77.7, 87.0)	0.936
Children under 6 months exclusively breastfed (%)	170	89.1 (80.9, 94.0)	96.2 (80.2, 99.4)	87.4 (77.6, 93.3)	0.165
Children 6–35 months with age-appropriate dietary diversity (%)	869	47.3 (42.2, 52.5)	44.4 (34.7, 54.5)	48.2 (42.3, 54.1)	0.523
Children 6–35 months with age-appropriate meal frequency (%)	869	66.7 (61.8, 71.2)	70.6 (62.2, 77.8)	65.5 (59.7, 70.8)	0.295

^a^ All values are means (95% CI) or percentage (95% CI), as indicated.

^b^ MPI score ≥ .33 is considered “poor.”

^c^ “Optimal IYCF practices,” based on continued breastfeeding, increased dietary diversity, and increased meal frequency based on child’s age range, is defined as ICFI = 6.

### Take-home ration coverage

**Fig 1:**
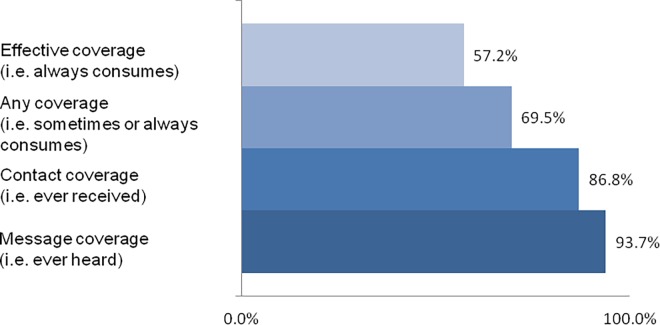
Bal Amrutham Coverage Indicators among Children 6–35 Months (N = 905).

The coverage of the take-home ration among children between 6 and 35 months is presented in Fig 1. Only 2% of the children below 6 months incorrectly received the take-home rations and exclusive breastfeeding prevalence was high. No significant difference in the coverage levels was found between rural and urban areas and therefore the results are not presented. The only difference between poor and non-poor groups was at the level of message coverage: Caregivers from poor households were less likely to have heard of Bal Amrutham than caregivers from non-poor households (88.6% versus 95.7%, p<0.05). Most caregivers of children under 6 months (80.8%) had already heard of Bal Amrutham, but only 2.2% had ever received it for the child.

### Coverage barriers

The main barriers to the coverage of the take-home rations were insufficient or depleted stocks at the AWC and intra-household sharing of the product. Regarding stock outages, 28% of caretakers reported that Bal Amrutham was out of stock at the AWC, and 39% reported that the AWC did not provide Bal Amrutham, which is a potentially a proxy response for stock outages. Difference in responses related to stock outages were not significantly different by MPI (poor vs. non-poor) or residence (urban vs. rural). In only 3% of the respondents, the belief that the product caused diarrhea or vomiting in the child was a barrier. This may be attributable to mixing the product with unsafe water or milk (which was done in 16% of the households).

The product was commonly shared among household members (71.0% of caregivers, 44.1% of other children under 5 years of age, 23.6% of older children or adolescents, and 25.1% of other adults in the household also consumed Bal Amrutham). The target children consumed smaller amounts than intended (45 grams of Bal Amrutham, instead of 83 grams). Despite these findings, the product still provided significant amounts of macro- and micronutrients to the target children: 22.0% of the energy, 34.3% of the protein, 15.5% of the fat, 51.3% of the iron, 22.9% of the vitamin A, 16.0% of the folic acid, 60.0% of the thiamine, 44.8% of the riboflavin, 38.5% of the niacin, 18.9% of the vitamin C, and 28.3% of the calcium recommended dietary allowances for Indian children. These intakes did not differ significantly between poor and non-poor children or between rural and urban households (data not shown).

Poor households were less likely to have visited an AWC in the past month (85.2% for poor households versus 92.8% for non-poor households, p<0.05). The main reasons mentioned for not going to the AWC every month were: (i) the distance from the household to the center or the cost of getting to center and (ii) the husband or family not allowing the caregiver to go to the center.

### Coverage drivers

The main drivers of the consumption of Bal Amrutham were: (i) the taste of the product, (ii) the fact that it is good for the children, and (iii) the fact that it is free. Moreover, the access to the ICDS program was high and a significant driver to coverage. In fact, all caregivers (100.0%) had heard of the AWC before, 98.1% had already been to an AWC, and 91.0% had been to an AWC in the past month.

### Coverage, need, and risk

The percentage met need and CRs are shown in Table 4. The results show a high percent met need in those at risk of poverty and poor IYCF practices. None of the CRs were significantly different from 1, indicating no differences in targeting between those at risk and those not at risk.

**Table 4:** Percent Met Need and Coverage Ratios of Bal Amrutham^a^.

Type of coverage	Risk group	Met need (%)^b^	Coverage ratio (mean)^c^
Message coverage	Poverty^d^	91.3 (80.4, 99.2)	0.94 (0.83, 1.01)
	Poor IYCF^e^	95.4 (92.2, 97.9)	0.98 (0.95, 1.02)
Contact coverage	Poverty	85.5 (72.5, 95.3)	0.96 (0.82, 1.06)
	Poor IYCF	88.5 (82.8, 93.5)	0.99 (0.92, 1.07)
Any coverage	Poverty	63.4 (47.9, 77.3)	0.90 (0.69, 1.11)
	Poor IYCF	67.4 (60.4, 74.3)	0.92 (0.82, 1.05)
Effective coverage	Poverty	52.4 (40.9, 64.6)	0.91 (0.71, 1.14)
	Poor IYCF	55.9 (49.0, 61.9)	0.95 (0.79, 1.14)

^a^ Results are presented for all children 6–35 months of age (N = 905) and are mean (95% CI) or percentage (95% CI), as indicated.

^b^ Met need is the estimated coverage in the at-risk group. Estimated using blocked weighted bootstrap estimation technique.

^c^ Coverage ratio is the ratio of coverage estimates in at-risk vs. not at-risk groups. Estimated using blocked weighted bootstrap estimation technique.

^d^ MPI score ≥ 0.33 is considered at risk of acute poverty.

^e^ ICFI score < 6 is considered poor IYCF.

## Discussion

The coverage of Bal Amrutham was high; nearly 70% of target children had consumed Bal Amrutham and about 58% of target children being the sole consumer of Bal Amrutham in their households. This coverage did not vary significantly between poor and non-poor households, which suggests that a household's socio-economic status is not a barrier to accessing Bal Amrutham. Furthermore, our study illustrates that the targeting of complementary take-home rations to children above 6 months of age via large-scale distribution systems can be done successfully with little "leakage" to children <6 months old, the period where exclusive breastfeeding is recommended. The main drivers of coverage were the high coverage of the ICDS program itself and more specifically the taste of Bal Amrutham, the caregivers’ perception as the product being good for the children, and the fact that the product was free. The IYCF practices of the children surveyed were generally poor, and highlights the need for improve the practice of continued breastfeeding and to increase dietary diversity and meal frequency in young children.

Results from the present survey showed high program performance: Access and utilization of the ICDS program’s services were high, as were all coverage measures. Results for the present study showed that 75% of the children between 6 and 35 months had received supplementary food in the past month, which is higher than the previous findings. A previous assessment conducted in 2005–2006 as part of the third National Family Health Survey (NFHS-3) showed that, in the State of Andhra Pradesh, 29% of the children under 36 months of age living in the catchment areas of AWCs received supplementary food in the past 12 months [17]. There are some differences between these two surveys: The previous survey was conducted in the whole state of Andhra Pradesh, while the present survey was conducted only in the part of Andhra Pradesh that is now the State of Telangana. Despite these differences, there have been large increases in the number of AWCs, which is the most likely reason for increase in coverage[18].

### Strengths and limitations of the study

The strengths of this study were that: (i) the surveyed population included children under 6 months of age and allowed assessment of early introduction (i.e., before 6 months) of complementary foods and, more specifically, of Bal Amrutham; (ii) the survey questionnaire was composed of standardized modules, which make the results of this survey comparable to other survey results; and (iii) both coverage and need were assessed, which allowed the analysis of the overlap between both indicators.

The principal limitation of the study is that the results are representative of the population living in the catchments areas of the AWCs and are therefore not generalizable to the whole population of Telangana. It is not known whether the sampling framework excluded vulnerable communities and households from the sampling frame. There is some evidence, however, to suggest that families who do not live in the catchment areas of AWCs are more likely to live in urban areas, have attained higher education levels, not be from any scheduled tribe or caste, and be wealthier [19]. The second limitation is that children over the target age range (35 months) were not surveyed and therefore results can make no inferences about households with older children.

### Generalizability

Despite the limitation of generalizability to the whole population, the results are highly relevant to the ICDS program: They show that the coverage within the AWC catchment areas is very high and that, in those areas, a high proportion of the need is met by the program. Results from this survey may be useful to other states, but caution should be made about generalizing to the country at large or to other states, as there is a lot of variability on the coverage of the ICDS program. In fact, already in 2005–2006, the ICDS program in the region of Andhra Pradesh was performing better than the national average, in part due to a high number of AWCs: Nationally, 33% of the children under 36 months of age living in the catchment area of an AWC received any service from the AWC in the past 12 months, which was 53 percentage points less than in Andhra Pradesh [3, 17]. The performance of the ICDS program varies widely between states [3] and depends mainly on the distribution and availability of funding for the ICDS program in each state and on the number of AWC that are in service [8].

Despite state-level differences, a number of barriers to coverage and utilization identified in this study are relevant to other programs distributing fortified take-home complementary foods nationally. Intra-household sharing of products targeted at young children, in particular, was a considerable challenge faced by the project. Similar finding have been observed in Malawi and Mozambique and the intra-household consumption of lipid-based nutrient supplements targeted at young children [19]. Use of "safe" drinking water or milk when preparing Bal Amrutham, though only occurring in ~15% of respondents, is likely a challenge that could be faced in other areas of India where there is little access to safe drinking water sources and water treatment products.

The results of this survey highlight that centralized production can be an effective strategy to increase nutrient intakes among young children. Centralized production has a number of advantages, including standardized production, stronger quality control standards, and the possibility of using advanced micronutrient formulations and production processes, as well as economies of scale [20]. These advantages likely favored the high coverage achieved in this project. However, it also has numerous challenges, such as high start-up capital requirements, long ramp-up period, high concentration of risk, lack of local economic empowerment, and lack of link with end beneficiaries. In efforts to improve the acceptability and utilization of the ICDS, as well as to promote local empowerment, the Supreme Court of India is promoting decentralization and passed an order in 2004 that supported the use of local women’s self-help groups, rather than commercial contractors, as providers of the supplementary food distributed through the ICDS program. Whether centralized or decentralized production is better will certainly be context-specific and dependent on several characteristics such as existing production capacity.

### Program recommendations and future research

Despite the coverage achieved, a number of challenges related to product distribution remain. Bal Amrutham is supplied by AP Foods to the ICDS program at the block level; where the program is then responsible for distributing the product to the AWC, and the AWWs are responsible for distributing the product to the beneficiaries. The results confirm that AWWs are distributing Bal Amrutham in appropriate amounts to the correct target beneficiaries; however, the supply of Bal Amrutham to AWCs was a problem. A large proportion (~30%) of mothers who had not received Bal Amrutham in the past month specifically gave stock outages at the AWC as a reason. A further analysis of the supply chain would allow the accurate identification of the key bottlenecks and determination of areas of improvement.

This study found that intra-household sharing was common, a phenomenon that has been encountered in a number of supplementary food distribution programs, including those in Niger[21], Brazil [22], India, and Pakistan [23], among many others. Greater intra-household food sharing is associated with poorer nutritional response to supplementary feeding[24]. Possible ways to address this include: (i) increasing consumer awareness by providing clear messages and education to ensure that only the target children receive the food and (ii) increasing the portion of the supplementary food to ensure that the target individual still consumes sufficient amounts of the food even if sharing takes place [25]. In the present study, although the product was shared among household members, the target children still consumed the product in quantities that provided between 16% and 60% of their requirements in macro- and micronutrients. What cannot be answered by the survey findings is what the rest of the diet looks like and what contribution the product makes to overall intakes in the diet. An additional study would need to be conducted to further assess the dietary intakes of the children. This study would also allow for the determination of other micronutrients that are missing from the diets of the target children, such as zinc [26] and vitamin B12 [27], and for the formulation of recommendations on how to improve the formulation of Bal Amrutham.

Despite some implementation challenges, our study illustrates that take-home rations can be successfully distributed through India's ICDS system with reported consumption by appropriately targeted children. This study was designed to estimate coverage and identify supply and demand issues that may limit both coverage and utilization. Accurate data of coverage and utilization of such interventions, as well as of their barriers and drivers, is needed to inform further program adjustments. Once these implementation issues have been adequately addressed, an impact evaluation may be useful to quantify effectiveness to improve nutritional status.

## Conclusions

The ICDS program is an important delivery channel for services to children in the State of Telangana. The supplementary food product, Bal Amrutham, distributed through the ICDS program to children between 6 and 35 months in Telangana is widely available, accepted, and consumed among the target population in the catchment areas of the AWCs. However, to maximize the potential for impact, supply chain issues, particularly ensuring continual supply at the point of distribution, should be addressed. Similarly, further education and potentially complementary strategies that would support successful targeting of the product to the intended child (6 to 36 months of age) in the home are needed.

## Supporting Information

S1 File STROBE Checklist. (DOCX)
